# Comparative analysis of primary *versus* relapse/refractory DLBCL identifies shifts in mutation spectrum

**DOI:** 10.18632/oncotarget.18502

**Published:** 2017-06-15

**Authors:** Danielle M. Greenawalt, Winnie S. Liang, Sakina Saif, Justin Johnson, Petar Todorov, Austin Dulak, Daniel Enriquez, Rebecca Halperin, Ambar Ahmed, Vladislav Saveliev, John Carpten, David Craig, J. Carl Barrett, Brian Dougherty, Michael Zinda, Stephen Fawell, Jonathan R. Dry, Kate Byth

**Affiliations:** ^1^ Oncology Innovative Medicines and Early Development, AstraZeneca R&D Boston, Waltham, MA, USA; ^2^ Translational Genomics Research Institute, Phoenix AZ, USA; ^3^ Center for Algorithmic Biotechnology, Institute of Translational Biomedicine, St. Petersburg State University, St. Petersburg, Russia; ^4^ Current address: Translational Bioinformatics, Bristol-Myers Squibb Company, Hopewell, NJ, USA

**Keywords:** DLBCL, R-CHOP, NGS, mutation, resistance

## Abstract

Current understanding of the mutation spectrum of relapsed/refractory (RR) tumors is limited. We performed whole exome sequencing (WES) on 47 diffuse large B cell lymphoma (DLBCL) tumors that persisted after R-CHOP treatment, 8 matched to primary biopsies. We compared genomic alterations from the RR cohort against two treatment-naïve DLBCL cohorts (n=112). While the overall number and types of mutations did not differ significantly, we identified frequency changes in DLBCL driver genes. The overall frequency of *MYD88* mutant samples increased (12% to 19%), but we noted a decrease in p.L265P (8% to 4%) and increase in p.S219C mutations (2% to 6%). *CARD11* p.D230N, *PIM1* p.K115N and *CD79B* p.Y196C mutations were not observed in the RR cohort, although these mutations were prominent in the primary DLBCL samples. We observed an increase in *BCL2* mutations (21% to 38% of samples), *BCL2* amplifications (3% to 6% of samples) and *CREBBP* mutations (31% to 42% of samples) in the RR cohort, supported by acquisition of mutations in these genes in relapsed compared to diagnostic biopsies from the same patient. These increases may reflect the genetic characteristics of R-CHOP RR tumors expected to be enriched for during clinical trial enrollment. These findings hold significance for a number of emerging targeted therapies aligned to genetic targets and biomarkers in DLBCL, reinforcing the importance of time-of-treatment biomarker screening during DLBCL therapy selection.

## INTRODUCTION

Non-Hodgkin’s lymphoma (NHL) is the most prevalent form of lymphoma, comprising an estimated 88% of 80,900 new lymphoma cases in 2015 [1]. Approximately 30-40% of new NHL cases are diffuse large B-cell lymphoma (DLBCL) [2], a heterogeneous form of NHL that can be further classified based on B-cell differentiation stages. A number of studies have explored the mutation spectrum of DLBCL by focusing on treatment-naive DLBCL. However, these studies provided variable estimates of the most prevalent gene mutations [3-6]. Furthermore, 40% of DLBCL patients still relapse after initial treatment with the R-CHOP (Rituximab-cyclophosphamide, doxorubicin hydrochloride, vincristine sulfate, prednisone) immunochemotherapy regimen, which is considered the standard of care (SOC) for DLBCL [7].

Clinical trials are underway to explore targeted agents against genetic drivers of DLBCL post R-CHOP failure, however until recently little has been known about the mutational landscape after immunochemotherapy treatment. Understanding the landscape is critical for development of novel targeted treatments for DLBCL for which clinical trial success depends on initial studies in patients who have failed SOC therapies. To put this into context, we identified 54 clinical studies of novel therapeutics in relapsed/refractory (RR) DLBCL that were opened during the sample collection period (2010-2015) for the samples evaluated in this study [8]. Of these, 38 studies involved novel small molecule inhibitors that included BCR-targeted agents, PI3k inhibitors, epigenetic modulators, proteasome inhibitors and immunomodulatory drugs. The remainder of the trials were primarily comprised of novel therapeutic antibodies and bio-similars. In the entire set we found sixteen trials where the trial design incorporated collection of DNA and/or RNA for biomarker assessment. The majority of these trials incorporated gene expression profiling for the purposes of cell of origin (COO) subtype identification, five trials included mutational analysis of specific genes, but only four trials included an intent to analyze the genomic landscape of DLBCL, including the cohort we analyzed and a recent study reported by Morin et al [9].

To gain a better understanding of the differences in somatic alterations between primary and post R-CHOP DLBCL we evaluated publically-available whole exome sequencing (WES) data from two published treatment-naïve studies [3, 4] (n=112). We then performed WES on core needle biopsies from 47 patients following 1-8 rounds of R-CHOP therapy[10]. Comparison of alterations and their context in these two datasets provides a deeper understanding of the somatic alteration spectrum of DLBCL and how this may change post R-CHOP. While we find similarities in the mutation spectrum of our cohort with previously published reports [9, 11-13] it is clear that the genomic nature of the patient pool differs between clinical studies and over time, underscoring the importance of evaluating RR tumors post treatment and prior to targeted therapy.

## RESULTS

FASTQ files from two independent studies, Pasqualucci *et al* [3] and Zhang *et al* [4], totaling 112 samples, were downloaded from dbGAP. WES was performed on core needle biopsies from 47 RR patients. All FASTQ files were processed through the same pipeline to align and call somatic variants. An average of 4.1 million reads mapped per sample in the Zhang study compared to an average of 1.3 million in the Pasqualucci study. Therefore, we found a much lower overall coverage in the Pasqualucci study, with a cohort average read depth of 15X compared to 30X in the Zhang study. However, these differences did not translate to large differences in the number of overall and novel variants identified across the two cohorts. Given the low coverage for the Pasqualucci study, we did not include this data in our copy number analyses. From the 47 RR samples, an average of 139 million reads per sample was mapped with a mean target coverage of 138X. Despite the different sequencing depths, there was not a large overall difference in the average number of somatic non-silent mutations observed between patients from the primary (average = 282) versus RR cohorts (average = 249).

We first explored the mutation frequencies in DLBCL associated genes in the 112 primary DLBCL samples [3, 4]. We observed *CREBBP, PIM1, TP53, BCL2, CARD11, TNFAIP3, EP300, EZH2, CD79B,* and *MYD88* to be mutated in greater than 10% of samples (Table 1A). Many of these genes have been identified as highly mutated in DLBCL in previous reports [3, 4, 14, 15]. Known mutations in DLBCL were also observed including *MYD88* p.L265P (8%), *EZH2* p.Y646F/N (9%), *CARD11* p.D230N (4%) and *CD79B* p.Y196C (3%) (Table 1B). Driver analysis was performed using OncodriveFM and OncodriveCLUST at https://www.intogen.org/ to identify genes with genetic variation that occur in three or more samples implicating a role in driving tumorigenesis [16]. This analysis confirmed that a number of these genes were significant drivers in our cohort (Q>0.01) including *EZH2, MYD88, CREBBP* and *TP53*. Other genes of interest found to be significant drivers through this analysis include *BCLAF1, NOTCH2, FAS, B2M, CDC27* and *SYK*) Notably this driver analysis also prioritized a number of likely false positive variant changes from genes in a ‘black list’ previously reported[17, 18] as artifacts of next-generation-sequencing and data processing, including *FRG1, NCOR1, PABPC1, USP17L and MUC20.*

**Table 1:** A. Primary vs RR mutation frequency B. by amino acid change. B. Primary vs RR mutation frequency B. by amino acid change.

A			B			
Gene Symbol	% Primary	% RR	Gene Symbol	AA Change	% Primary	% RR
*CREBBP*	31.25%	41.67%	*MYD88*	L265P	8.04%	4.17%
*8CL2*	20.54%	37.50%	*EZH2*	Y646N	4.46%	
*TP53*	21.43%	35.42%	*TP53*	R248Q	4.46%	
*MYD88*	11.61%	18.75%	*EZH2*	Y646F	4.46%	4.17%
*B2M*	8.04%	18.75%	*CARD11*	D230N	3.57%	
MYC	9.82%	14.58%	*P1M1*	5188N	3.57%	2.08%
*EP300*	13.39%	12.50%	*PIM1*	K115N	3.57%	
*BTK*	5.36%	12.50%	*CD798*	Y196C	2.68%	
*P1M1*	22.32%	10.42%	*CREBBP*	R1446C	2.68%	
*CARD11*	16.07%	10.42%	*EZH2*	Y6465	2.68%	
*EZH2*	13.39%	10.42%	*CREBBP*	51680de1	2.68%	6.25%
*CD798*	12.50%	6.25%	*CREBBP*	R1446H	2.68%	2.08%
*BCL6*	7.14%	6.25%	*MYD88*	5219C	1.79%	6.25%
*JAK1*	1.79%	6.25%	*TP53*	R273C	1.79%	2.08%
*MALT1*	1.79%	4.17%	*BCL2*	P59A	0.89%	8.33%

**Figure 1:**
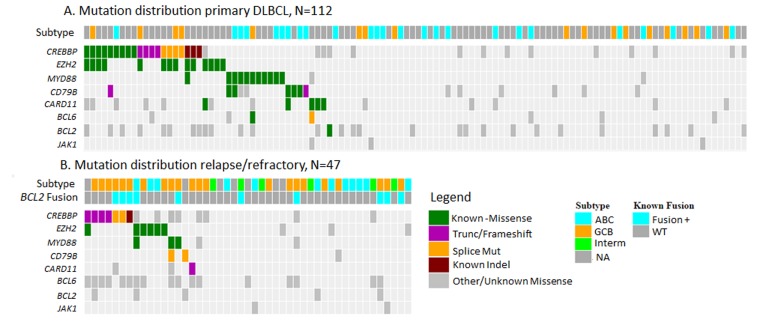
Type and distribution of mutations identified in **A.** primary DLBCL samples and **B.** relapse/refractory to R-CHOP DLBCL samples. Mutations represented in COSMIC and known to have functional consequences are represented as Known.

A number of gene and amino acid level mutation frequencies differed between the primary versus RR datasets (Table 1; Figure 1). Overall frequency of *MYD88* increased from 11.6% in primaries to 18.8% in RR DLBCL, however frequency of the p.L265P mutation dropped from 8% in primaries to 4% in the RR cohort, and p.S219C increased from 1.8% in primaries to 6.3% in the post treatment dataset. In *BCL2,* we found an increase in 5’ UTR mutations with 16.7% of the RR cohort compared to 6.3% of the primary DLBCL cohort. The overall mutation frequencies of *CARD11, PIM1* and *CD79B* decreased in the RR samples. Notably, a number of known DLBCL variants [3, 4, 14, 15] present in the primary cohorts were absent in the RR cohort, including *CARD11* p.D230N, *PIM1* p.K115N and *CD79B* p.Y196C mutations. These results were visually inspected in IGV [19, 20] and no reads were found to support the mutations in any of our samples, despite sufficient depth of coverage and quality. We did identify two novel splice site mutations in *CD79B* at the beginning of the ITAM (immunoreceptor tyrosine-based activation motif) domain, however their functional effects are unknown. Driver analysis found that *CREBBP*, *BCL2AF1, TP53, EZH2 and MYD88* remained significant (Q < 0.01) in the RR dataset. *B2M, SYK* and *NOTCH2* were not found to be significant, but *FUBP1* emerged as a significant driver in the RR dataset. Two nonsense and a frameshift mutation were found in *FUBP1* (Far Upstream Element (FUSE) Binding Protein 1) in the RR cohort. *FUBP1* regulates MYC expression by binding to a single-stranded FUSE upstream of the *MYC* promoter. *FUBP1* mutations have also been identified in oligodendrogliomas [21]. When comparing to other recent published studies in RR DLBCL specifically we did find differences in the reported frequency of mutations, beyond the hotspots noted. Morin et al[9] identified hotspot mutations in *STAT6* and *FOXO1* in a similar cohort of RR DLBCL patients*.* A single RR patient was found to have a *STAT6* D419G in our RR cohort, no re-current mutations were found in *FOXO1*. *NFKBIE* and *NFKBIZ* were also found to be frequently mutated in the Morin dataset [9]. We found 1 RR subject with the reported *NFKBIE* frameshift deletion that has also been identified aggressive CLL previously [22], but no *NFKBIZ* variants were found in our RR cohort.

We then evaluated relative consensus copy number changes across the two cohorts. *REL* was found to be the most recurrently-amplified gene in primary and RR cohorts, consistent with previous reports[12] . No other significant amplifications were found through analysis of the primary tumor dataset. In the RR samples, we identified three significantly amplified peaks (q < 0.05) impacting gene regions for *REL, BCL2* and *MYC*. A fourth amplification was found at 13q32.1, but no cancer census genes [23] were annotated in or neighboring the peak. *BCL2* amplification frequency increased from 3% in the primary samples to 9% in the relapse samples. The increase in *BCL2* and *MYC* amplifications observed in the RR cohort is consistent with reports that *BCL2* and *MYC* amplification or translocation are associated with worse prognosis in DLBCL patients [24-29]. Interestingly a previous report of recurrent copy number alterations in RR DLBCL did not identify regions on chromosome 8 (*MYC*), 13 or 18 (*BCL2*) [12].

**Figure 2:**
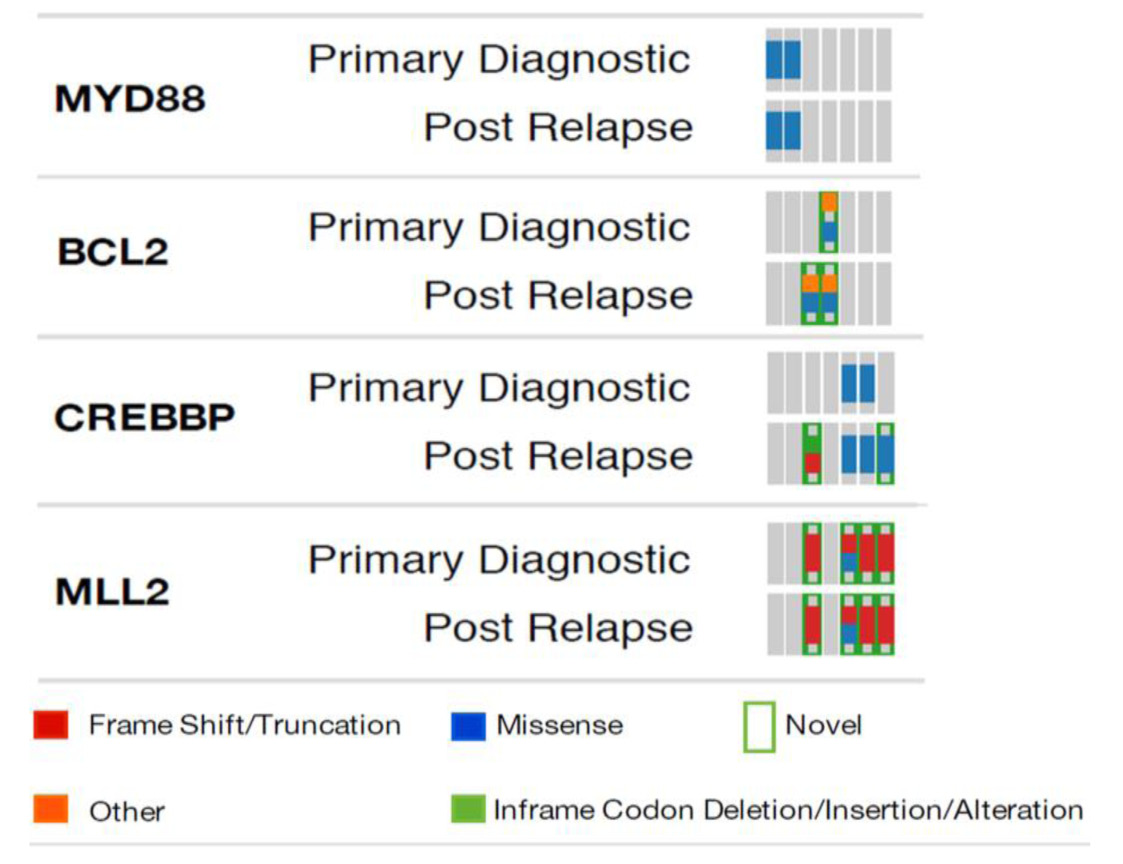
Mutation comparison in pre-post treatment biopsies from the same patient

We had access to primary tumor FFPE samples from 8 of the 47 RR subjects that allowed us to evaluate mutations that emerged post RCHOP therapy. Consistent with the mutation frequencies we observed to increase in our independent cohort comparisons, we found new *CREBBP* and *BCL2* mutations emerge in the matched RR samples (Figure 2).

DLBCL is a heterogeneous tumor type and tumor subtypes derived from gene expression signatures have been used to define the COO of DLBCL [30, 31]. Activated B-cell (ABC) and germinal center B-cell (GCB) COO subtypes have also been characterized by their mutation profiles. To ensure that the mutational shifts observed were not due to subtype composition of the cohort, we separately evaluated the mutation frequencies of each subtype where data was available. Subtype classifications were available for 42 of the primary samples, 21 ABC and 21 GCB [3, 4], and 33 of the RR cohort [32]. Within the RR cohort 13 samples (39%) were annotated as ABC and 21 (63%) as GCB. We found that the enrichment for *CREBBP* and *BCL2* mutations in the GCB subtype was greater. This enrichment in GCB was also observed in the primary DLBCL cohorts. One sample which had been subtyped as ABC was found to have mutations in *MYD88* (S219C), *EZH2,* and *BCL2,* which are normally associated with the GCB subtype [3, 33]. Previous analysis of gene expression signatures has also found that the COO subtype signature does not change after treatment [32]. However, to our knowledge this analysis has not been performed at the DNA level.

## DISCUSSION

To gain a deeper understanding of the mutational spectrum of DLBCL we analyzed WES data from two primary DLBCL cohorts and an R-CHOP RR cohort. Characterizing the similarities and differences in the mutational landscapes of these cohorts is essential to understanding the impact of treatment on tumors, as well as understanding the genomic context of patient populations who are not served by standard therapies. Although the overall mutation rate post R-CHOP did not differ significantly, we did identify a near doubling in frequency of mutations in *CREBBP* and *BCL2* between primary and RR cohorts, which are supported by differences between paired primary and RR samples from several patients. In addition to an increase in the number of mutations in *BCL2*, we also observed an overall increase in *BCL2* amplification frequency.

Analysis of our RR cohort led to the identification of *FUBP1* as a possible driver specific to the RR cohort. *FUBP1* regulates MYC expression, which has been shown to be a marker of worse prognosis in DLBCL. Nonsense mutations in *FUBP1* may affect the regulation of MYC in these patients. Further analysis of *FUBP1* and its role in DLBCL post SOC is necessary to understand this finding.

Our findings provide support that although matched pre-treated samples are often not available for analysis, cohort analysis indeed provides insight into mutational changes that arise during or after treatment. Caution should be paid, however, to differences arising due to sampling. Overall we found many differences when comparing to other similar whole exome sequencing studies on post-SOC DLBCL. Strikingly, we found no *CD79B* hotspot mutations in our RR cohort. We also found differences in novel hotspots and recurrently mutated genes from recent RR DLBCL studies, including those reported in *STAT6*, *FOXO1* and *NFKBIZ* [7]^,^ [9]. This finding may be a consequence of sampling and trial enrollment at the institution where these samples were collected. It is also important to consider variation introduced as a result of differences in sequencing and variant calling techniques between cohorts. We took steps to control for this is in our own study by reprocessing all raw data through the same computational pipeline, however this could also explain discrepancies with recent publications on similar cohorts. It should be noted that cohort mutational landscape differences in DLBCL have also been observed in primary DLBCL studies [4].

Overall, our findings highlight shifts in mutational composition across DLBCL patient populations with respect to R-CHOP treatment. Continued molecular characterization of cohorts in the treatment naïve and post treatment settings is necessary to improve therapeutic strategies for RR patients. For this reason, we caution the use of historical data to predict the genomic nature of future trial cohorts, particularly in the RR setting. We also note that there is a need to better understand the genomic landscape of RR tumors, which is often not considered in current trial designs. Implementing molecular diagnostics based on such characterizations will be critical for ensuring the success of targeted treatments in DLBCL.

## MATERIALS AND METHODS

Fourty Seven post R-CHOP fresh frozen core needle biopsies were obtained from DLBCL patients, as defined by Cheson [34], who were refractory or relapsed following 1-8 cycles of R-CHOP. This RR cohort is a subset of the patients described by Flinn et al [10], from whom biopsies were available after COO assessment described by Veldman-Jones et al previously [32]. Of the 47 patients, diagnostic FFPE biopsies were available for 8 patients and matched blood samples were available for 14 patients.

### Genomic DNA extractions

Genomic DNA was extracted from fresh frozen tissue using one of two procedures. Genomic DNA for 9 samples (Cohort A) was isolated from fresh frozen tissue using the AllPrep DNA/RNA Mini Kit (Qiagen) and the Bullet Blender BlueTM (Next Advance). Genomic DNA extractions for the additional 38 samples (Cohort B) from FFPE tissue on the QIAcube (Qiagen) using the AllPrep DNA/RNA FFPE Kit.

### Library construction and next generation sequencing

For Cohort A and B, and 8 FFPE tumor DNAs (pre-R-CHOP) exome libraries were constructed using SureSelect Human All Exon V5+UTR baits (Agilent) and KAPA Biosystem’s Library Preparation Kit using the manufacturer’s “with bead” protocol. For Cohort C exome libraries were constructed using SureSelect Human All Exon V5 (51Mb, no UTR) baits (Agilent) and the TruSeq DNA Library Preparation Kit (Illumina). Libraries were paired-end sequenced on the Illumina HiSeq platform using TruSeq SBS (sequencing by synthesis) reagents (Illumina).

### External datasets

FASTQ files from two independent studies, Pasqualucci *et al* [3] and Zhang *et al* [4], totaling 112 samples, were downloaded from NCBI’s (National Center for Biotechnology Information) dbGaP (Database of Genotypes and Phenotypes) [35], accessions phs000328.v2.p1 and phs000573.v1.p1.

### Data processing and analysis

Alignment and variant calling was performed within the BCbio framework (https://github.com/chapmanb/bcbio-nextgen). Reads were aligned to the hg19 human reference genome assembly using BWA [36], no realignment or recalibration was performed. Duplicate reads were removed from final BAM files using Samblaster [37]. Variants were called using VarDict [38], with thresholds of minimal allowed read support of 3, minimal mean position in reads of 5, minimal mean base quality phred score of 25, and minimal mean mapping quality score of 10.

Mutations were annotated using SnpEff [39] according to the NCBI RefSeq’s gene model. Known somatic and germline actionable (i.e. known as responsive to a targeted therapy) mutations with allele frequency ≥ 2.5% were prioritized. Common germline SNPs, specifically SNPs not reported in COSMIC [40], but reported in dbSNP [41] and annotated mostly as benign or likely benign according to ClinVar [42], or having a global minor allele frequency > 0.0025 in TCGA, were removed from downstream analysis. Additionally, variants were filtered by cohort frequency: novel variants present in ≥ 40% and ≥ 10 samples with average allele frequency < 15%, and any other variant present ≥ 75% and ≥ 10 samples, were considered too common to be functional. Germline variants found in the 14 matched normal samples were also excluded from downstream analysis. A comprehensive annotated mutation file is included as supplemental (Supplementary Tables 1 and 2).

Seq2C (https://github.com/AstraZeneca-NGS/Seq2C/wiki) was used to estimate gene copy-number variation by comparing normalized mean gene coverage across samples in a cohort. Four cohorts were processed separately: two external datasets, both using regions from Agilent SureSelect Human All Exon V4 capture BED file; 38 RR samples using Agilent SureSelect Human All Exon V5 BED capture file; and 37 RR samples using Agilent SureSelect Human All Exon V5+UTR capture BED file. Outlier genes with low coverage were removed using a 3x upper/lower quartile threshold, and filtered data were segmented with the DNAcopy [43] package using default settings in the R statistical software (https://www.r-project.org/). GISTIC2.0 [44] was implemented to identify consensus copy number alterations using the following settings: gene.gistic = yes, amplifications.threshold = 0.2, deletions.threshold = 0.2, join.segment.size = 4, qv.thresh = 0.25, remove.X = yes, cap.val = 1.5, confidence.level = 0.75, broad.length.cutoff = 0.98, max.sample.segs = 2500, arm.peel = no.

## SUPPLEMENTARY MATERIALS TABLES






