# Supplementary material for: Comparative analysis of primary versus relapse/refractory DLBCL identifies shifts in mutation spectrum
Source: Oncotarget. 2017 Jun 15;8(59):99237–44. doi: 10.18632/oncotarget.18502 (PMC5725088; doi:10.18632/oncotarget.18502)
Supplement: Supplementary file 1 [file oncotarget-08-99237-s001.pdf]

# Comparative analysis of primary *versus* relapse/refractory DLBCL identifies shifts in mutation spectrum

## SUPPLEMENTARY MATERIALS

**For Supplementary Tables see in Supplementary Files.**
